# Retinoblastoma-binding protein 2 (RBP2) is frequently expressed in neuroendocrine tumors and promotes the neoplastic phenotype

**DOI:** 10.1038/oncsis.2016.58

**Published:** 2016-08-22

**Authors:** E C Maggi, J Trillo-Tinoco, A P Struckhoff, J Vijayaraghavan, L Del Valle, J S Crabtree

**Affiliations:** 1 Department of Genetics, Louisiana State University Health Sciences Center, New Orleans, LA, USA; 2 Stanley S. Scott Cancer Center, Louisiana State University Health, New Orleans, LA, USA; 3 Departments of Medicine and Pathology, Louisiana State University Health, New Orleans, LA, USA

## Abstract

Neuroendocrine tumors (NETs), which can have survival rates as low as 4%, currently have limited therapeutic interventions available highlighting the dire need for the identification of novel biological targets for use as new potential drug targets. One such potential target is retinoblastoma-binding protein 2 (RBP2), an H3K4 demethylase whose overexpression has been linked to cancer formation and metastasis in non-endocrine tumor types. We measured RBP2 mRNA and protein levels in enteropancreatic NETs by measuring RBP2 in matched human normal and NET tissue samples. Further, proliferation, migration, invasion and colony formation assays were performed in the physiologically relevant NET cell lines βlox5, H727 and QGP-1 to understand the role of RBP2 and its demethylase activity on end points of tumorigenesis. Our data indicate a strong correlation between RBP2 mRNA and protein expression in NET specimens. RBP2 was overexpressed relative to tissue-matched normal controls in 80% of the human tumors measured. *In vitro* studies showed RBP2 overexpression significantly increased proliferation, migration, invasion and colony formation, whereas knockdown significantly decreases the same parameters in a demethylase-independent manner. The cell cycle inhibitors p21 and p57 decreased with RBP2 overexpression and increased upon its depletion, suggesting a regulatory role for RBP2 in cellular proliferation. Taken together, our results support the hypothesis that the aberrant overexpression of RBP2 is a frequent contributing factor to tumor formation and metastasis in enteropancreatic NETs.

## Introduction

Neuroendocrine tumors (NETs) arise from neuroendocrine tissues in the lung, pancreas, gut and bowel, and pose a significant threat because of their high metastatic potential. The 5-year survival rate can be as low as 4% in patients with certain subtypes of poorly differentiated NETs^1^ and at present, there are limited therapeutic options. Surgery is the only curative approach, but is effective only if the tumors are removed prior to metastasis.^2^ Although there have been moderate successes with somatostatin analogs, mTOR inhibitors and tyrosine kinase inhibitors (sunitinib) as therapy in some subtypes of NETs, there still remains a dire need for novel, effective pharmaceutical treatments.^3, 4, 5^ One such potential target is retinoblastoma-binding protein 2 (RBP2/KDM5A/JARID1A), an important cell cycle regulator associated with cancer development and prognosis in non-NET tumors.^6, 7, 8^ RBP2 is an H3K4 demethylase capable of forming multiple different protein complexes with binding partners including RB, CSL/RBP-Jk and others.^9, 10, 11, 12, 13, 14^

In non-NETs, RBP2 directly activates cell growth through its demethylase activity by decreasing the expression of cell cycle inhibitors, and indirectly affects cell cycle arrest by forming complexes with multiple proteins to regulate transcriptional activation.^8, 11, 15, 16^ A recent report demonstrates that inhibiting RBP2 production in a pancreatic NET mouse model halts tumor formation;^17^ however, this study did not demonstrate a causative role for or a mechanistic impact of RBP2 overexpression. Therefore, we investigated the mechanistic role of RBP2 overexpression in NET formation. To do this, we studied the levels of RBP2 in human samples by quantitative real time PCR and immunohistochemistry, and performed proliferation, migration, invasion and colony formation assays in NET cell lines with or without ectopic RBP2 expression. In addition, we examined the effect of RBP2 knockdown or overexpression on cell cycle inhibitors and have identified p57 as a previously unknown downstream target of RBP2.

## Results

### RBP2 expression is elevated in NETs

**Figure 1:**
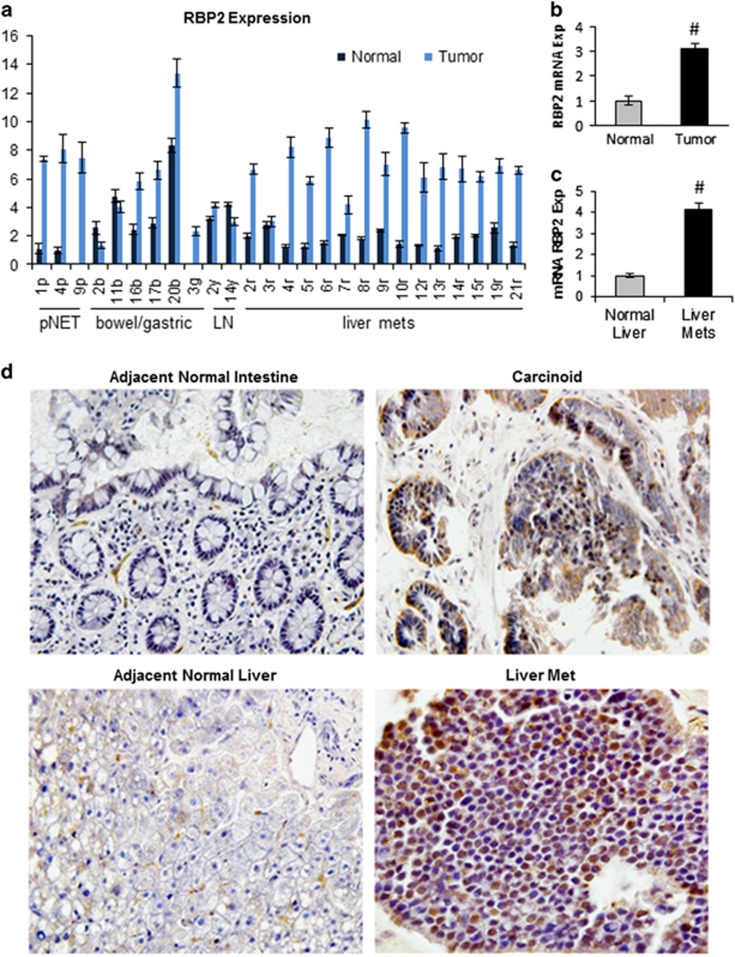
RBP2 is overexpressed in human NETs. (**a**) Relative RBP2 mRNA expression levels in NET tumor samples and matched normal tissues taken at time of surgery with the lowest normal tissue value set to 1. The sample number (*x* axis) corresponds to the patient and the letter to the tissue type where p=pancreatic, b=bowel, g=gastric, y=lymph node, and r=liver. (**b**) Average RBP2 mRNA elevation in normal tissue and NETs with the normal tissue average set to 1 to calculate fold increase of RBP2 and (**c**) average RBP2 mRNA elevation in normal liver and liver mets with the normal tissue average set to 1 to measure fold increase of RBP2 compared to normal (^#^*P*<0.005). (**d**) Immunohistochemistry on representative tumor and normal samples (× 200 original magnification) showing an increase in the level of RBP2 protein in the tumors. The small intestine carcinoid is a primary tumor and the liver is a metastasis from the same patient. Sections were developed with diaminobenzidine (RBP2 expression is brown), and counterstained with hematoxylin.

RBP2 was significantly elevated at the RNA level in 20 out of 25 human tumor samples compared with matched normal tissue (Figure 1a), with an average upregulation greater than threefold (Figure 1b). This was most evident in the group of liver metastases with 14/15 showing significant RBP2 elevation and 8/15 with increases between 6 and 10 fold (4.1 average fold increase; Figure 1c). The observation of significantly increased levels of RBP2 in liver metastases suggests a role for RBP2 in the metastatic tumors. Immunohistochemistry yielded similar results as RNA analysis (Figure 1d), showing an increase in RBP2 protein in neuroendocrine primary tumors and secondary metastatic sites compared with adjacent normal, non-tumor tissue. In addition, immunohistochemistry showed that the subcellular localization of RBP2 varies depending on the degree of neuroendocrine differentiation as determined by chromogranin staining (Figure 1d and Supplementary Figure 1). RBP2 was detected in the cytoplasm of the well-differentiated tumor cells (Figure 1d, carcinoid), but in the nucleus of poorly differentiated cells (Figure 1d, liver met).

### Generation of stable cell lines and knockdown of RBP2

**Figure 2:**
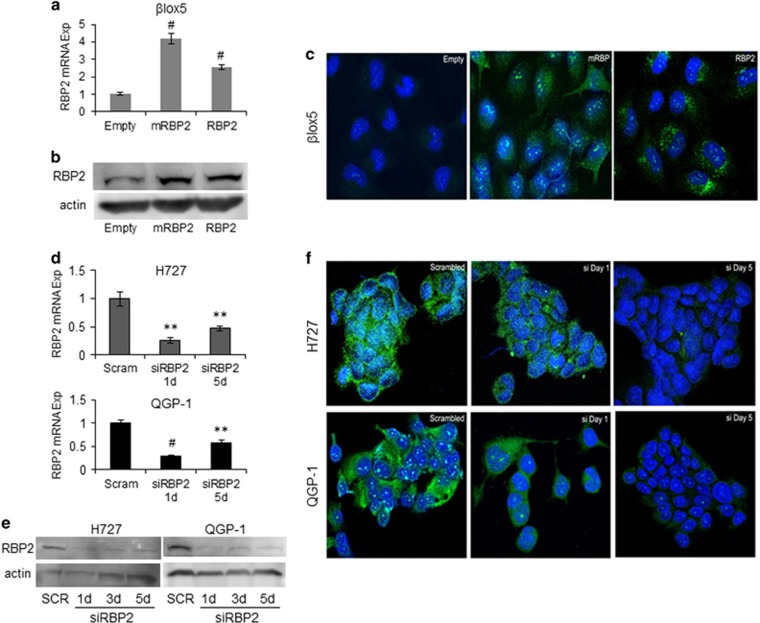
RBP2 overexpression in βlox5 and knockdown in H727 and QGP-1 cell lines. RBP2 mRNA levels were measured in βlox5 cell lines stably expressing either empty vector (Empty), enzymatically dead mutant RBP2 (mRBP2) or normal RBP2 (RBP2) by (**a**) qRT–PCR, (**b**) western blot and (**c**) immunofluorescence. Alexa-Fluor 488-RBP2 is green and cells were counterstained with DAPI. H727 or QGP-1 cells were transiently transfected with 10 pmol of either scrambled (scram/SCR) or RBP2 siRNA for 1–5 days and analyzed for RBP2 expression levels by (**d**) qRT–PCR, (**e**) western blot and (**f**) immunofluorescence. (**P<0.02, ^#^*P*<0.005).

To further investigate the role of RBP2 in NETs, stable overexpressing cell lines were created in the βlox5 parental cell line, which contains minimal endogenous expression of RBP2, and is derived from normal pancreatic beta cells transformed with SV-40 T-antigen. The βlox5 cells were individually transduced with empty vector, a mutant RBP2 with a point mutation in the active site (mRBP2; a correctly folded, yet enzymatically dead protein that retains the ability to bind partner proteins and chromatin^12^), or wild-type RBP2. Expression levels of RBP2 and mRBP2 were increased two- to threefold compared with empty vector as measured by quantitative real time PCR (Figure 2a). Western blotting (Figure 2b) and immunofluorescence for RBP2 was performed on the βlox5 stable cell lines and overexpression was observed in both the cytoplasm and nucleus of the mRBP2 and RBP2 stable cells (Figure 2c). This increase correlates with the physiological expression levels and pattern of expression observed in human NETs compared with the adjacent normal tissue (Figures 1a and b).

Conversely, to further understand the role of RPB2 in NET formation, we transiently knocked down RBP2 in the NET cell lines H727 (derived from lung carcinoid NET) and QGP-1 (derived from somatostatinoma pancreatic NET), which both have high levels of RBP2. We reduced the expression of RBP2 by transiently transfecting RBP2 or control small interfering RNAs (siRNAs) (Figures 2d–f), with knockdown being maintained for up to 5 days post transfection, thereby demonstrating knockdown for the duration of subsequent proliferation assays and target gene expression. RBP2 mRNA is reduced at least 50% in the H727 cells and QGP-1 cells achieved a 40% knockdown across all 5 days.

### RBP2 enhances proliferation by targeting p21 and p57

**Figure 3:**
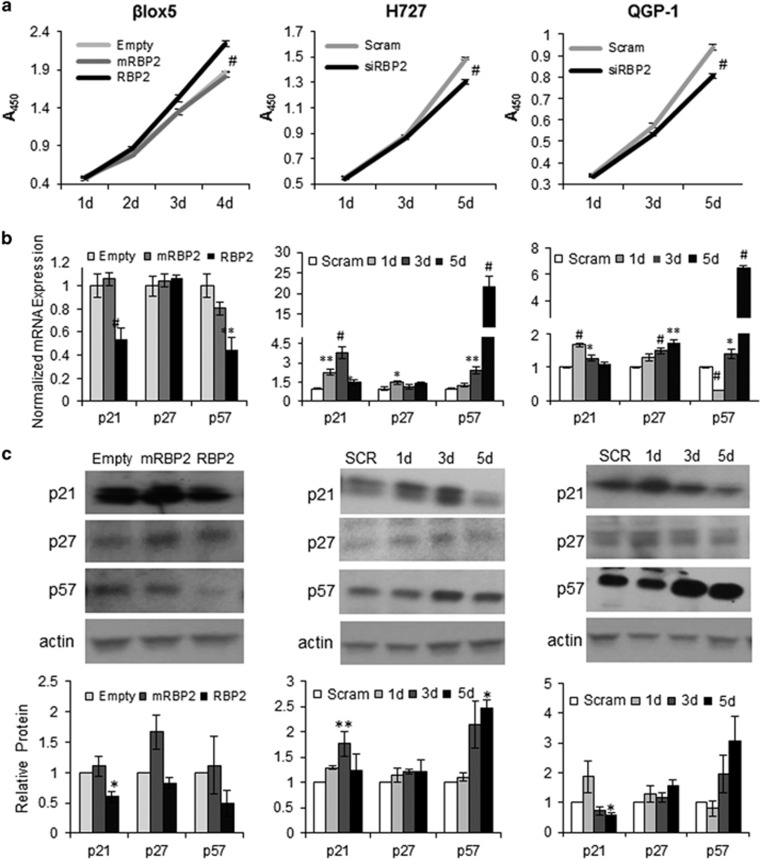
Analysis of cell lines with overexpression and knockdown of RBP2. (**a**) Stable βlox5 cells overexpressing RBP2, mRBP2 or empty vector were assessed for proliferation every day for 4 days. Similar proliferation assays were performed every other day for days for the H727 and QGP-1 cells lines that were transiently transfected with scrambled si or RBP2si, with the assay beginning 24 h after transfection. (**b**) mRNA levels of p21, p27 and p57 in stable overexpressing βlox5 cells, and H727 and QGP-1 transiently transfected with RBP2 siRNAs. (**c**) Representative western blots for the same targets in cell lines as above in (**b**) and protein quantitation via densitometry of p21, p27 and p57 resulting from three separate experiments using actin for normalization. Significance was measured relative to the respective control (*P<0.05 **P<0.02 ^#^*P*<0.005).

To measure the impact of RBP2 on cell proliferation, we measured cell density as a function of time using the stable overexpressing cell lines and the transient RBP2 knockdown cells to modulate the levels of RBP2. From these studies, we determined that overexpression of RBP2 but not mRBP2, significantly increased proliferation in βlox5 cells (Figure 3a), whereas knockdown of RBP2 in H727 and QGP-1 cell lines showed significant decreases in cell proliferation (Figure 3a). These results suggest that RBP2 enhances proliferation of neuroendocrine cells and the enzymatic activity may be necessary for these effects.

Based on previously published RBP2 studies,^8, 18, 19^ which demonstrate the effect of RBP2 on downstream targets, we measured the effect of knockdown and overexpression of RBP2 on the known targets p21 and p27 in addition to the previously unpublished target p57 (Figures 3b and c). We examined the effect of overexpression of RBP2 and mRBP2 in the βlox5 cells and found that at both the RNA and protein levels, p21 and p57, but not p27, were significantly decreased with wild-type RBP2 overexpression, but not with mRBP2 overexpression, consistent with our proliferation results. In the tumor cell lines H727 and QGP-1, we measured the effect of knockdown over the course of 5 days and measured differences in target gene expression. In the H727 cells, p21 increased quickly and by day 5 began to decrease, whereas p57 increased significantly by day 5. As in the βlox5 cells, there was little to no change in p27. In the QGP-1 cells a similar trend was measured with the exception of an initial drop in p57 levels on day 1 followed by a robust increase (Figure 3c). Taken together these results demonstrate that RBP2 affects cellular proliferation in NETs by targeting p21 and p57, but not the anticipated target, p27.

**Figure 4:**
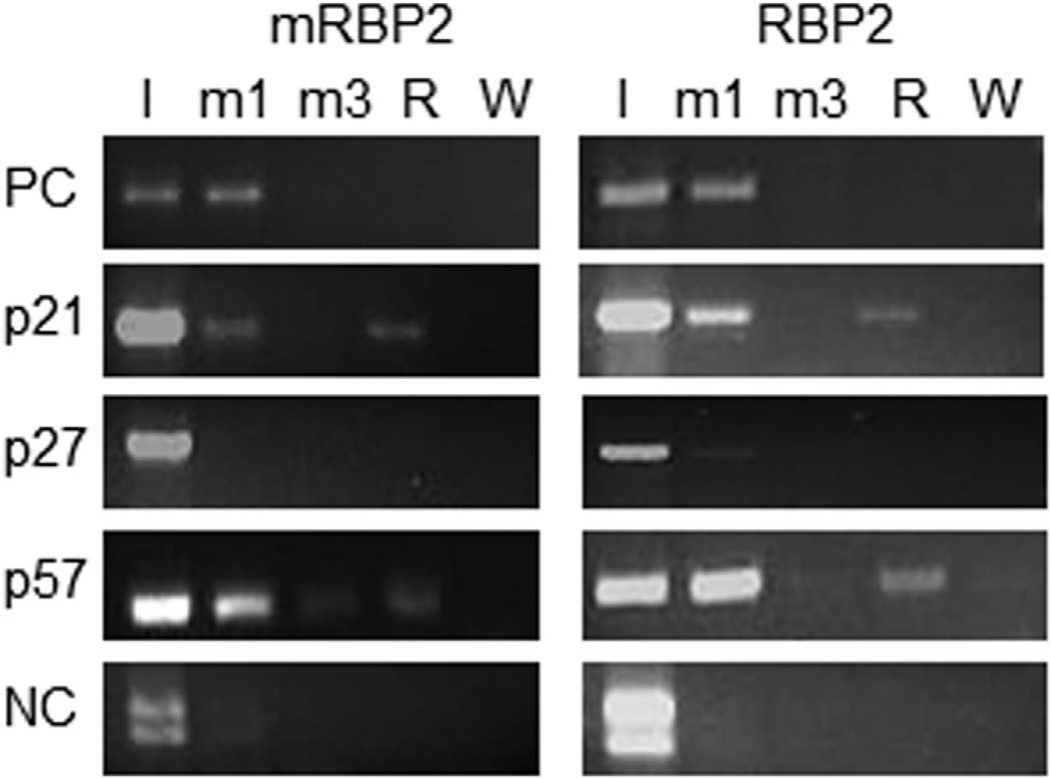
ChIP analysis of downstream RBP2 target genes. Chromatin IP from βlox5 cells overexpressing either mRBP2 or RBP2. Antibodies against either mono or tri-methylated H3K4 or RBP2 were used for ChIP. Conventional PCRs was run using primer sequences from the regulatory region of p18 (as a positive control for monomethylation ChIP; PC), p21, p27, p57 or an unrelated region of the genome as a negative control (NC). I–input, m1–H3K4me1 ChIP, m3–H3K4me3 ChIP, R–RBP2 ChIP, W–water control for PCR.

To confirm that the target gene changes result from direct binding of RBP2 to the respective promoters, we performed Chromatin Immunoprecipitation (ChIP) studies using antibodies that recognize RBP2, and mono- or tri-methylated H3K4 in the βlox5 RBP2 and mRBP2-overexpressing cell lines. We then analyzed the immunoprecipitates for p18 (positive control), p21, p27 and p57 promoter DNA by conventional PCR (Figure 4). Consistent with the above results, we found direct binding of RBP2 to the p21 and p57 promoters in the presence of H3K4 monomethylation. Although qualitative in nature, these studies suggest that RBP2 demethylase activity, which acts only on di- or tri-methylated histones, could be affecting the histone methylation levels at the promoter to impact gene expression. Consistent with our mRNA and protein results in which we observe no RBP2-dependent changes in p27, there was minimal binding of RBP2 to this promoter.

### RBP2 affects biological properties relevant to metastasis

**Figure 5:**
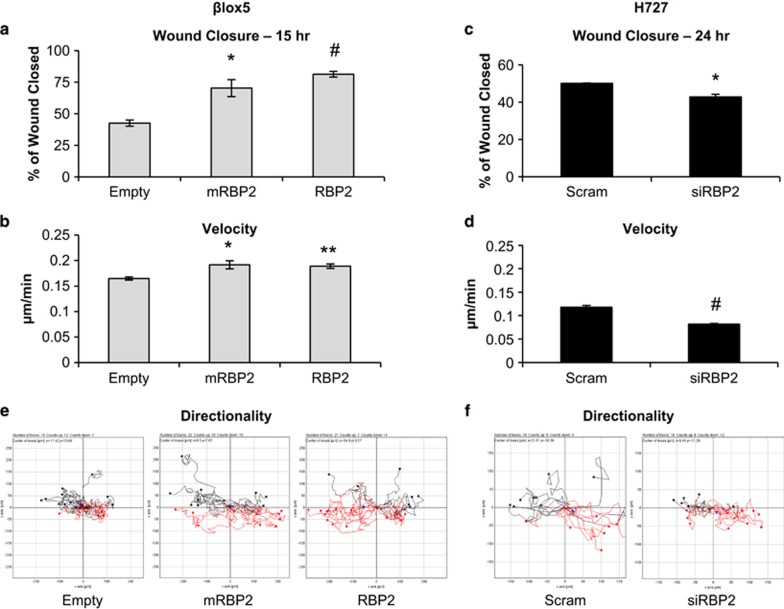
Migration assays in βlox5 and H727 cell lines. A scratch was made in confluent (**a**) stable βlox5 cell lines and (**c**) transiently transfected H727 cell lines and the average percent wound closure was calculated 15 or 24 hrs later, respectively, from three independent experiments. (**b** and **d**) Average velocity was calculated from 60 individual cell trackings for each condition from (**b**) overexpression and (**d**) knockdown. (**e** and **f**) Directionality plots of mobility data from 20 individual cells (10 from each side of the scratch) showing direction and distance from origin of individual cells in each condition. (*P<0.05 **P<0.02 ^#^*P*<0.005).

In addition to increased proliferation, changes in migratory ability are also important for the development of metastatic disease. Therefore, we performed migration (wound healing) assays on the stable βlox5 and transiently transfected H727 cells. QGP-1 cells were technically not amenable to this assay because they grow in clumps instead of as a monolayer. We found that upon overexpression of either mRBP2 or RBP2 the wound healed significantly faster than empty vector (Figure 5a), which resulted from an increase in the average velocity (Figure 5b). Conversely, knockdown of RPB2 resulted in a significant decrease in wound healing and cellular migration velocity (Figures 5c and d). An examination of the individual cell tracings, which illustrates the distance and directionality of individual cells, also demonstrated that RBP2 overexpression increased, whereas knockdown decreased the distance and directionality of cell movement (Figures 5e and f). Combined, these data indicate RBP2 is capable of changing the motility of cells. The enzymatic activity of RBP2 is not critical for this function, in contrast with the transcriptional effects described above.

**Figure 6:**
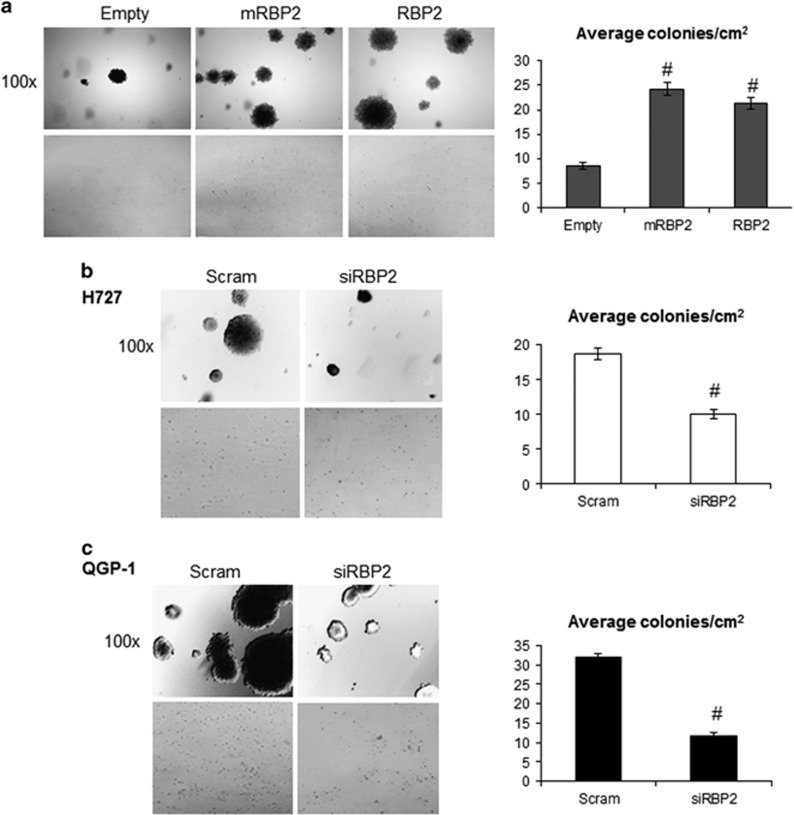
Colony formation with RBP2 overexpression and knockdown. (**a**) βlox5 cells stably expressing either empty vector (Empty), enzymatically dead mutant RBP2 (mRBP2), or wild-type RBP2 (RBP2) were used for colony formation assays and (**b**) H727 and (**c**) QGP-1 cells were transiently transfected with either a scrambled siRNA (Scram) or an RBP2 siRNA (siRBP2) and followed 24 hrs later by colony formation assays. Colonies were stained with crystal violet after 3 weeks and pictures were taken at × 100 magnification or with a standard hand held camera. In addition, colonies present in 25 × 1 cm^2^ fields were counted and an average number of colonies per cm^2^ was calculated for each condition. (^#^*P*<0.005).

The ability of cells to grow in the absence of a solid support is considered one of the hallmarks of transformation. Therefore, we performed colony formation assays in soft agar with our cell lines. We measured significant increases in the number and size of colonies with both mRBP2 and RBP2 overexpression in βlox5 (Figure 6a), with an average increase of 2.5 fold for both compared with empty vector. Consistent with our overexpression results, we also measured a significant decrease in the number and size of colonies upon RBP2 knockdown in both H727 and QGP-1 cells (Figures 6b and c). Combined with the results presented above, this observation strengthens the idea that modest RBP2 overexpression, as seen in human tumors, contributes to tumor growth and metastasis.

**Figure 7:**
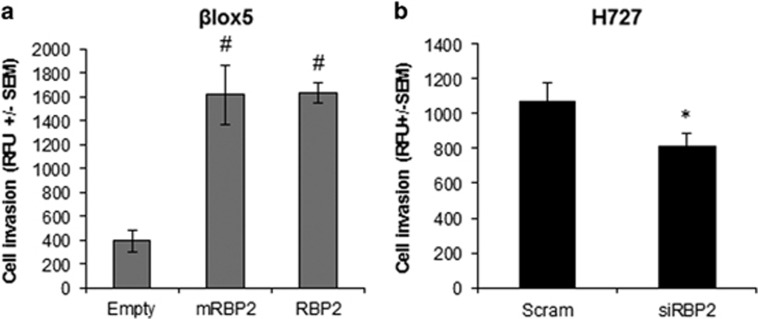
Invasion assays of βlox5 stable cells and H727 transiently transfected cells. Relative fluorescent units (RFU) were measured from (**a**) βlox5 stable overexpressing cell lines or (**b**) stable RBP2-overexpressing H727 cells transiently transfected with either scrambled (Scram) or RBP2 siRNA having invaded through Matrigel in an invasion assay. RFUs were measured on a bottom-reading plate reader and average RFU was calculated for each experimental condition. (*P<0.05 ^#^*P*<0.005).

To determine how RBP2 affected the invasiveness of these cells, we performed invasion assays in our βlox5 stable cell lines (Figure 7a) and H727 transient RBP2 knockdown (Figure 7b). We found no invasion with the QGP-1 cells and therefore were unable to use this line for these assays (data not shown). RBP2 and mRBP2 stable expression promoted a fourfold increase in invasion relative to the empty vector control (Figure 7a), whereas stable knockdown resulted in a modest, yet significant decrease (Figure 7b). As with migration, we observed no difference between the mRBP2 and RBP2 stable cell lines suggesting that histone demethylase enzymatic activity is not required for invasion.

## Discussion

Treatment options for patients with metastatic NETs are limited. Debulking surgery remains the first line therapy; however, due to the metastatic potential of these tumors it is rarely curative, highlighting the dire need for new potential drug targets. Traditional chemotherapy is generally ineffective and patient management often encompasses only symptom palliation.^20^ Previous studies demonstrated RBP2 overexpression in non-NETs;^19^ however, we are the first group to show overexpression of RBP2 in NETs and their metastases. In this study, we identified and quantified RBP2 overexpression in 20 out of 25 human NET samples, or 80% of the tumors measured. The percentage of liver metastases with increased expression of RBP2 (93%) and the higher degree of elevation (threefold in all tissue types, fourfold in liver mets) further indicates RBP2 could be having a role in metastasis. This result is supported by our data demonstrating that the level of RBP2 expression correlates directly to changes in tumorigenic end points of proliferation, migration, invasion and colony formation. Therefore, we conclude that RBP2 contributes to NET tumor progression and may serve as a potential therapeutic target or as a useful starting point to identify downstream targets for drug development.

Of particular interest is our observation that although proliferation depends on RBP2's demethylase activity by impacting p21 and p57 expression levels, we found no statistical difference between the RBP2- and mRBP2-overexpressing cells on the effects on migration, invasion and colony formation. These observations are particularly provocative because they suggest that the demethylase inhibitors that are currently under development by several laboratories may not be entirely effective as NET therapeutics, especially in the majority of patients that present with advanced metastatic disease. Furthermore, the independence of migratory, invasive and colony formation ability from enzymatic activity suggests that the effects of RBP2 on these phenotypes may depend on specific binding partners present in a particular cellular milieu rather than on demethylase-dependent control of target genes expression. A recent paper focusing specifically on the role of RBP2 in metastasis of breast cancer cell lines found that the demethylase activity was not required to increase tenascin C expression but that the C- and N-terminal protein domains were required, suggesting the importance of binding partner interactions for disease progression.^6^ This study is consistent with our data in its implication that interaction with putative, yet unidentified binding partners is an important mechanism by which RBP2 influences metastasis.

Perhaps, the most interesting and unexpected observation was the nearly universal difference in RBP2 localization in well-differentiated versus poorly differentiated neuroendocrine cells in tumor specimens. Several studies suggested that among the tissues of origin there are often two different types of NETs that can arise, one the more conventional, slow growing carcinoid and the other a much more aggressive and poorly differentiated tumor.^21, 22^ Our results suggest that the differences in the behavior of these two tumor types may depend at least in part, on RBP2 subcellular localization: cytoplasmic in well-differentiated tumor cells, and nuclear in poorly differentiated tumor cells. This result is significant because subcellular localization may affect the ability of RBP2 to act as a histone demethylase as well as the potential binding partners that mediate its demethylase-independent effects.

We are the first group to identify p57 as a target gene of RBP2 in NETs. Previous reports identified several different RBP2 target genes including the cell cycle regulators p16, p21 and p27 in non-NET gastric and lung cancer. Consistent with these studies, we found p21 is a direct target of RBP2 regulation in NET tumor cell lines.^8, 18, 19^ However, unlike the previous studies in non-NET cells, we found no RBP2-dependent change in p27 expression in neuroendocrine cells, suggesting RBP2 may interact with different targets in a tissue or cell type-specific manner. Consistent with this notion, we observed a strong inverse correlation between p57 and RBP2 protein levels, as well as RBP2 binding to the p57 promoter, demonstrating p57 is a direct RBP2 target. This result is especially important because of a growing body of work highlighting the role of p57 repression in the development and spread of cancers.^23, 24^ Combined with our results, these published studies provide strong support that RBP2 promotes the development of NETs, in part, through the repression of p57. Furthermore, our novel p57 results are also intriguing as decreased levels of p57 serve as a prognostic indicator for patient outcome in non-NET cancers.^24^ Given that our results demonstrate a direct regulation of p57 expression by RBP2, it is highly likely that RBP2 can also be used as a prognostic marker in NETs. Furthermore, identifying p57 as a direct downstream target of RBP2 in NETs makes it a viable target for therapy, and drugs currently exist to increase p57 expression.^25, 26^

In very preliminary xenograft studies in nude mice (*n*=3 for each condition) we see the importance of RBP2 in tumor growth *in vivo* as well (data not shown). Subcutaneous xenografts from βlox5 cells overexpressing RBP2 result in a significant increase in tumor volume (*P*<0.02) 9 days after subcutaneous injection compared to βlox5 cells containing empty vector. Further, knockdown of RBP2 with siRNAs in H727 cells resulted in a significant decrease in tumor volume (*P*<0.005) 9 days after injection when compared with cells transfected with scrambled siRNA. In conclusion, the results presented here are the first to dissect the role of RBP2 in the development of NETs and to establish that the overexpression seen in patient tissue samples contributes to tumor formation and metastasis. We have identified p57 as a novel RBP2 target and shown that RBP2 enzymatic activity is required for some biological effects (for example, suppression of p57 expression) but not others (for example, effects on migration) in physiologically relevant cell lines. Together this information may be useful in development of novel NET therapeutics.

## Materials and methods

### Human tumor samples

De-identified human tumor samples and matched normal tissue (when available) were obtained from the surgical team of Dr Eugene Woltering (LSUHSC Endocrinology, Kenner, LA, USA). Tissue was placed in RNALater (Qiagen; Valencia, CA, USA) immediately after surgical removal. RNA was isolated from a portion of the sample using the RNEZ. Total RNA Isolation kit from Omega Biotek (Norcross, GA, USA). Remaining sample was divided and either placed in formalin for immunohistochemistry or frozen at −80C in RNALater for future use. Samples were collected with informed consent on a research tissue collection protocol (Woltering, PI, USA) approved by the Louisiana State University Health Sciences Center Institutional Review Board.

### Immunohistochemistry

Formalin-fixed, paraffin-embedded tissues were microtome-sectioned to a thickness of 4 μm, placed on electromagnetically charged slides (Fisher Scientific; Waltham, MA, USA), and stained with hematoxylin & eosin for routine histologic analysis. Immunohistochemistry was performed using the Avidin–Biotin–Peroxidase complex system, according to the manufacturer's instructions (Vectastain Elite ABC Peroxidase Kit; Vector Laboratories, Burlingame, CA, USA). Following antigen retrieval, slides were washed with phosphate-buffered saline (PBS) and blocked in PBS/0.1% bovine serum albumin containing 5% normal goat serum for 2 h at room temperature, then incubated overnight with primary anti-KDM5A (RBP2) rabbit polyclonal antibody (1:500, Sigma-Aldrich; St Louis, MO, USA) or anti-chromogranin A mouse monoclonal (Dako, 1:500; Carpinteria, CA, USA). The following day, slides were incubated with biotinylated secondary antibodies, developed using a diaminobenzidine substrate, counterstained with hematoxylin, and mounted with Permount. Images were collected at × 200 and × 600 magnification using an Olympus BX61 (UIS2 optical system) microscope equipped with a high resolution Olympus DP72 camera and CellSense image capture software.

### Stable cell line generation

The βlox5^27^ parental cell line (a kind gift from Dr Eric Lazartigues; mycoplasma-free) was stably transduced with either empty vector (pBabe-puro), enzymatically dead RBP2 expression vector (pBabe-puro/HA-FLAG-RBP2 H483A),^12^ or an RBP2 expression plasmid (pBabe-puro/HA-FLAG-RBP2) using the retroviral transduction system.^28^ Cells were maintained in 1.5 ug/ml puromycin. All expression plasmids were purchased from Addgene (Cambridge, MA, USA) and viral vectors were kindly provided by Dr Andrew Hollenbach.

### Immunofluorescence

H727^29^ (ATCC #CRL-5815, mycoplasma-free) and QGP-1^30^ (JCRB Cell Bank #JCRB0183, Japan; mycoplasma-free) cells transiently transfected with either a scrambled siRNA or RBP2 siRNA were seeded in two-well chamber slides from Thermo Scientific (Waltham, MA, USA) and grown to 80% confluency then washed with PBS, fixed with 70% ethanol for 3 min, then rinsed with PBS. Cells were incubated with a rabbit polyclonal anti-KDM5A(RBP2) overnight at room temperature. After rinsing with PBS, an Alexa-Fluor 488 anti-rabbit secondary antibody was incubated for 1 h at room temperature in the dark. Finally slides were rinsed with PBS, mounted with a DAPI containing, aqueous-based mounting media (Pro-Long Gold antifade, Invitrogen; Carlsbad, CA, USA), and visualized using an Olympus FV1000 confocal microscope. This was also done in stable βlox5 cells plated directly into two-well chamber slides.

### Proliferation assays

Cell metabolic activity (through the NADH-mediated reduction of a tetrazolium salt into formazan dye) was measured as a surrogate for cell number using the Cell Counting Kit-8 from Dojindo Molecular Technologies (Rockville, MD, USA) per manufacturer's instructions. For stable βlox5 cells 10 000 cells were seeded per well of a 24-well plate and measured every 24 hrs for 4 days. For transiently transfected H727 and QGP-1 cells 20 000 cells were seeded per well and measured every 48 h beginning on day one and continuing for 5 days, all conditions were performed in triplicate. For the transient transfections, Lipofectamine 2000 was used with 10 pmol of either scrambled or RBP2 siRNA to transfect 100 000 cells/well (all reagents from Life Technologies (Carlsbad, CA, USA)). A plasmid expressing green fluorescent protein (GFP) was co-transfected to assess transfection efficiency. Transfection efficiencies >80% were routinely achieved.

### RT–PCR

Total RNA was isolated using the RNEZ. Total RNA Isolation kit from Omega Biotek from either stable cell lines or from parental cells transiently transfected as above with RBP2 siRNA at days 1, 3 and 5. RBP2 (Hs00231908_m1), p21 (Hs00355782_m1), p27 (Hs01597588_m1), p57 (Hs00175938_m1) and RNA18S5 (Hs03928985_g1) were assessed using commercially available Taqman primer/probe sets from Life Technologies using a Bio-Rad CFX96. All experiments were performed in triplicate, with three biological replicates and normalized to RNA18S5.

### Western blots

Protein was isolated using RIPA Buffer from Santa Cruz Biotechnology (Dallas, TX, USa) with added Halt protease inhibitors (Thermo Scientific) from the stable cell lines and from RBP2 knockdown experiments at the same time points as used in RT–PCR. For target proteins p21, p27 and p57, westerns were run by standard procedures on 4–20% tris-glycine gels and transferred with the iBlot system from Life Technologies as previously described.^31^ Blots were probed using the following primary antibodies: anti-p21 Waf1/Cip1 (1:1000; Cell Signaling #2947; Danvers, MA, USA), anti-p27 (1:100; Santa Cruz Biotechnology #sc-528), anti-p57 Kip2 (1:1000; Cell Signaling #2557) and anti-βactin (1:3000; Cell Signaling #4967). Secondary antibody was horseradish peroxidase-labeled goat anti-rabbit IgG (1:5000; Perkin Elmer, Waltham, MA, USA) and the blots were developed with the ECL detection system (Thermo Scientific). RBP2 western blots were run by standard procedures as above with the following exceptions: proteins were separated using 8% tris-glycine gels and were transferred to membranes using submerged, tank transfer in Prosieve EX transfer buffer (Lonza, Basel, Switzerland). Primary antibody was anti-RBP2/KDM5A (Abcam #ab78322, 1:1000 for βlox5 and QGP-1 cells, Abcam #ab70892, 1:500 for H727 cells, Cambridge, MA, USA) and secondary antibody for these blots was horseradish peroxidase-labeled donkey anti-mouse IgG (Abcam #ab205724) or goat anti-rabbit IgG (Abcam #ab97501). All blots were performed on at least three biological replicate experiments and all three blots were used for densitometric analyses using Image J software.

### Chromatin Immunoprecipitation

The protocol for the Millipore ChIP Assay Kit (Billerica, MA, USA) was followed with minor changes. In brief, a confluent 10 cm dish of βlox5 RBP2 or mRBP2-overexpressing cells was scraped in cold PBS containing protease inhibitors (Halt Protease Inhibitor Cocktail, Thermo Scientific) and cross-linked with 1% formaldehyde, followed by the addition of glycine. Cells were washed 4 × with cold PBS containing protease inhibitors and resuspended in 400 μl SDS Lysis Buffer and 600 μl ChIP Dilution Buffer. Cells were immediately sonicated with 7 × 10 s bursts with a 20 s rest on ice in between sonications. After centrifugation the samples were pooled and the Millipore protocol was resumed with addition of 3.6 ml ChIP dilution buffer/ml sonicated lysate and preclearing with salmon sperm/bead slurry for 2 h. Before precipitation a 1 ml aliquot was taken from the sonicated, precleared sample and stored at –20 °C to be used as the input control. The following antibodies were used for the precipitation overnight at 4 °C: anti-JARID1A (RBP2) XP rabbit monoclonal (1:200, Cell Signaling), anti-H3K4me3 mouse monoclonal (1:200, Abcam) and anti-H3K4me1 rabbit polyclonal (1:200, Abcam). The following day, input samples were removed and decross-linking steps were performed simultaneously with the pulldown samples. The pulled down DNA was purified prior to PCR using the Qiagen PCR Purification Kit. A 1/10 dilution of input DNA was used for all PCRs which were run using DreamTaq Master Mix (Life Technologies) for 45 cycles per manufacturer instructions. ChIP Primer Sequences: p18 (CDKN2C) Forward: 5′-CATTTTGACCACTGGGTGCAT-3′ Reverse: 5′-ACTTCGGCAACCAAGAAATG-3′^31^ p21 (CDKN1A) Forward: 5′-CAGCTGCCGAAGTCAGTTCCT-3′ Reverse: 5′-CACCTGTGAACGCAGCACACA-3,^8^ p57 (CDKN1C) Forward: 5′-CCTGCTGGAAGTCGTAATCC-3′ Reverse: 5′-CACGATGGAGCGTCTTGT-3′,^32^ Negative Control (region of chromosome 14 with no known genes) Forward: 5′-GTTGTTGGATTTGGCTTGCT-3′ Reverse: 5′-GGACCAGATGGCATCATAGC-3.^31^ IP experiments were performed in triplicate, with three biological replicates.

### Migration assays

βlox5 stable or H727 transiently transfected cells were seeded in a 12-well plate and grown to confluency. A P-20 pipette tip was used to scratch the cell lawn and the progress of wound closure was tracked for 15 h (βlox5) or 24 h (H727) using the Olympus IX81 microscope with photographs taken every 1–2 h. Percentage of wound healed was quantitated by Image J software and three scratches were averaged to produce the graphs. In addition, Image J was used to track 20 individual cells from each experiment for directionality and velocity. For knockdown experiments, the cells were transfected 24 h before the start of the migration assay by following the same procedure as described for proliferation assays.

### Invasion assays

Invasion assays were performed per kit instructions using the BD BioCoat Matrigel Invasion Chamber Assay (Catalog No. 354480; Bedford, MA, USA). In brief, 2.5 × 10^4^ βlox5 stable cells (empty, mRBP2 and RBP2) or H727 RBP2-overexpressing cells that were transiently transfected with either scrambled or RBP2 siRNA were seeded in the wells in serum free media. 10% fetal bovine serum containing media was placed in the bottom chamber as a chemoattractant. The chambers were incubated for 48 h for βlox5 and 72 h for H727 then the media was removed from top and bottom and they were placed in 4 μg/ml Calcien AM solution (BD Biosciences, San Jose, CA, USA) for 1 h. The plate was then read at 949/517 nm (Ex/Em) on a bottom-reading fluorescent plate reader. Results were analyzed using Microsoft Excel. Images were taken at × 100 magnification using an Olympus IX71 microscope.

### Colony formation assays

βlox5 stable cell lines, and H727 and QGP-1 RBP2 transient knockdown cells were used. A bottom layer of media and 0.75% agarose was poured in a 10 cm dish and a top layer of media, 0.36% agarose and 167 000 cells was poured on top. Additional media was added to the top and colonies were allowed to form for 3-4 weeks with additional media being added as needed. The stable βlox5 cell lines were grown in media containing 1.5 μg/ml puromycin to ensure continued RBP2 overexpression. For the knockdown experiments H727 and QGP-1 cell lines were seeded into 6-well dishes and transfected as previously described using scrambled and siRBP2. After 24 h the cells were trypsinized and used in soft agar 10 cm dishes as above. The colonies were stained with 0.04% crystal violet and images were takes at × 100 using an Olympus IX71 microscope and a Nikon COOLPIX S80. Colonies were counted in an overall area of 25 cm^2^ and average colony per cm^2^ was calculated.

### Statistical analyses

Quantitative real time PCR data were analyzed by Student's *t*-test using GraphPad Online (www.graphpad.com/quickcalcs/ttest1). All other data were analyzed by Student's *t*-test using Excel. *P<0.05, **P<0.02, ^#^*P*<0.005. Data shown are mean±s.e.m.
