# Supplementary figures and images for: Retinoblastoma-binding protein 2 (RBP2) is frequently expressed in neuroendocrine tumors and promotes the neoplastic phenotype
Source: Oncogenesis. 2016 Aug 22;5(8):e257–. doi: 10.1038/oncsis.2016.58 (PMC5007832; doi:10.1038/oncsis.2016.58)

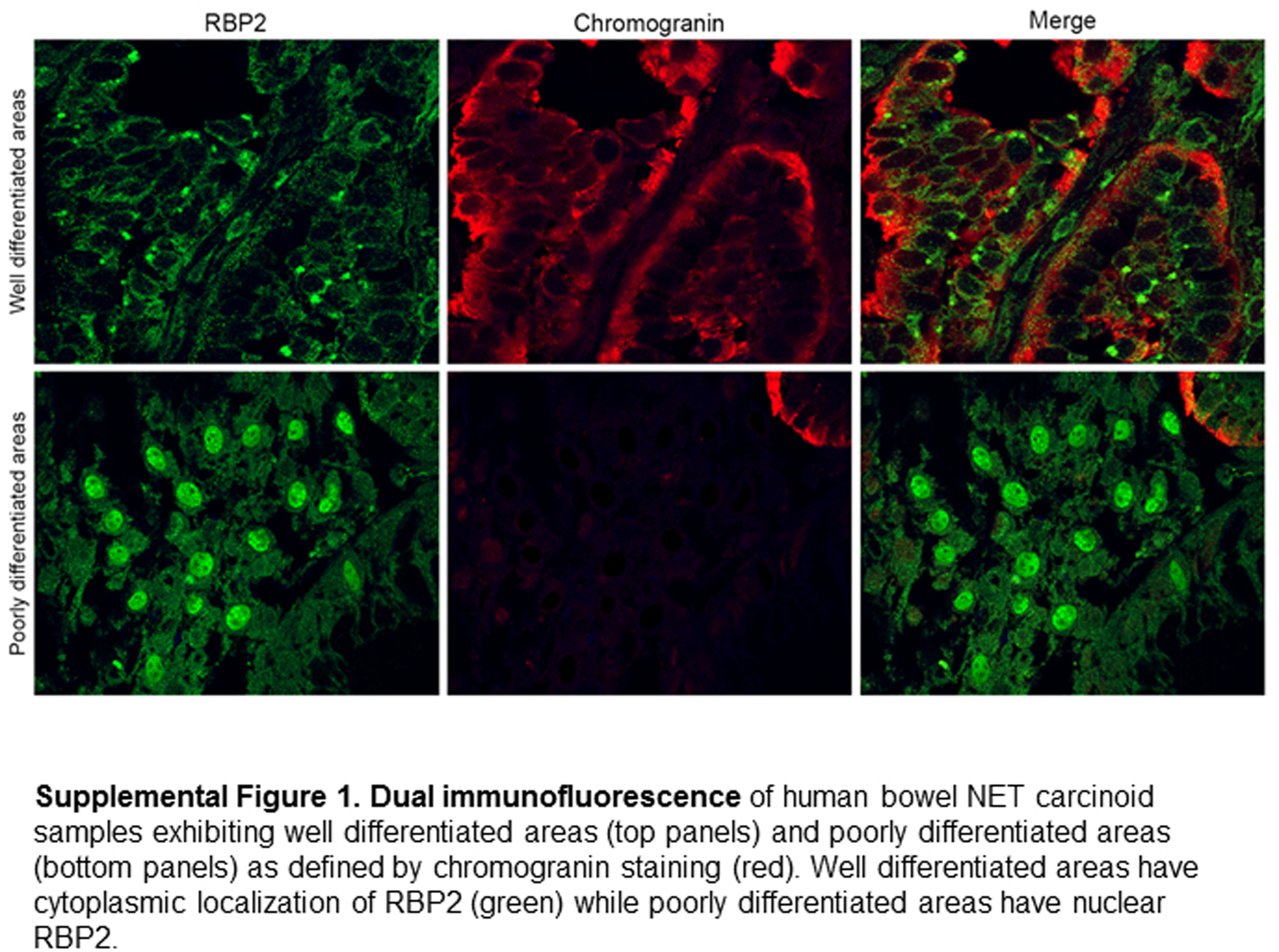

Supplement: Supplementary Figure 1 [file oncsis201658x1.tif]
